# Supplementary material for: Informal Employment and Poor Mental Health in a Sample of 180,260 Workers from 13 Iberoamerican Countries
Source: Int J Environ Res Public Health. 2022 Jun 27;19(13):7883. doi: 10.3390/ijerph19137883 (PMC9265366; doi:10.3390/ijerph19137883)
Supplement: Supplementary file 1 [file ijerph-19-07883-s001.zip › ijerph-1713128-supplementary.pdf]

Supplementary Table S1. Definition of employment condition by questionnaire.

Criteria	Country	Survey	Target and question	Employment condition	
				**Formal**	**Informal**
**Social security**	Argentina	National Survey of workers on Conditions of Employment, Work, Health and Safety	**Employees except for domestic workers:** Do you have a retirement discount for that job?	1. Yes	2. No
			**Domestic workers:** For that job, do you receive retirement contributions?	1. Yes	2. No
			**Self employed and employers:** Regarding retirement payment...	1 You pay as "monotributista"<br>2. You pay as a social "monotributista"<br>3 You pay as a freelancer<br>4. You contribute to a provincial or professional fund	5. You don't pay it because you don't have enough money to contribute<br>6. You don't pay it because the retirement would be very low<br>7.You don't not pay it because the retirement system is unreliable<br>8.You don't pay it for another reason
	Peru	National Survey on Working Conditions, Safety and Health	**All**: Do you have a discount, do you contribute, are you affiliated with, or registered in any retirement system (ONP/AFP)?	1. Yes	2. No
	Central America	II Central American Survey of Working and Health Conditions	**All**: Are you currently contributing to any retirement, unemployment or disability insurance, or social security or insurance fund?	1. Yes	2. No
**Health insurance**	Brazil	National Health Survey	**All**: Do you have any private, company or public medical health plan? Who is the holder of the medical health plan (single or main)?	1. Yes; Holder	1. Yes; Not holder<br>2. No
**Contract**	Chile	National Quality of Life and Health Survey	**All**: In your main job, do you have a written employment contract?	1 Yes, I signed.	2 Yes, but I haven't signed<br>3 I do not have a contract<br>4 I do not remember or do not know if I have signed
	Mexico	National Household Survey	**Employees**: Do you have a written contract in your main job?	1. Yes	2. No
	Spain, Portugal	European Working Conditions Survey	**Employees**: What type of contract do you have in your main paid job?	1 Permanent contract<br>2 Temporary contract<br>3 Contract with a temporary employment agency<br>4 Apprenticeship, training or internship contract	5 No contract

Supplementary Table S2. Population distribution by questionnaire, country and sex.

	Questionnaire and country						*continues*
	GHQ -12						
	**Costa Rica**	**El Salvador**	**Guatemala**	**Honduras**	**Nicaragua**	**Panamá**	**Peru**
	**N (%)**	**N (%)**	**N (%)**	**N (%)**	**N (%)**	**N (%)**	**N (%)**
**Men**	**766 (66.9)**	**637 (66.4)**	**785 (58.4)**	**779 (57.3)**	**674 (63.9)**	**921 (66.7)**	**1629 (53.5)**
**Employment condition**							
Formal	518 (62.3)	171 (16.3)	132 (12.0)	139 (14.3)	221 (24.0)	488 (43.9)	541 (37.1)
Informal	248 (37.7)	466 (83.7)	649 (88.0)	636 (85.7)	453 (76.1)	429 (56.1)	1071 (62.9)
**Labor relationship**							
Employee	417 (53.1)	317 (35.8)	288 (30.5)	342 (45.6)	279 (42.8)	435 (43.2)	921 (58.9)
Self-employed or employer	348 (46.9)	320 (64.2)	497 (69.6)	437 (54.4)	395 (57.2)	486 (56.8)	708 (41.1)
**Occupational category**							
Non-manual	388 (20.1)	287 (18.1)	354 (27.5)	232 (17.5)	276 (19.8)	243 (20.8)	759 (50.5)
Manual	378 (79.9)	348 (81.9)	431 (72.5)	546 (82.5)	396 (80.2)	416 (79.3)	870 (49.5)
**Age, years**							
under 24	90 (8.5)	90 (11.4)	151 (28.2)	98 (23.4)	95 (20.4)	139 (17.0)	351 (20.8)
25-44	351 (44.9)	312 (37.8)	357 (36.9)	331 (39.9)	347 (55.0)	431 (45.0)	716 (44.5)
45-64	270 (37.2)	172 (30.5)	221 (27.7)	261 (26.5)	191 (22.1)	284 (32.4)	415 (26.2)
≥65	55 (9.4)	63 (20.3)	56 (7.2)	89 (10.2)	41 (2.5)	67 (5.6)	147 (8.5)
**Education achieved**							
Less than primary school	16 (7.0)	27 (11.0)	53 (10.0)	62 (8.7)	66 (11.4)	25 (5.4)	83 (4.3)
Primary school	263 (50.5)	159 (38.4)	326 (48.9)	433 (57.3)	227 (37.9)	170 (24.6)	258 (14.6)
Secondary school	335 (33.3)	351 (42.6)	319 (33.2)	223 (29.2)	227 (35.9)	522 (55.9)	833 (52.3)
Higher education	152 (9.3)	100 (8.0)	87 (7.9)	61 (4.9)	154 (14.8)	204 (14.2)	455 (28.8)
**Marital status**							
Married or co-habiting	475 (66.8)	420 (63.6)	485 (60.1)	564 (67.7)	427 (62.5)	502 (54.3)	944 (56.4)
Single, divorced or widowed	291 (33.2)	217 (36.4)	298 (39.9)	215 (32.3)	245 (37.6)	419 (45.7)	684 (43.6)
**Women**	**737 (33.1)**	**737 (33.1)**	**725 (41.6)**	**728 (42.7)**	**826 (36.1)**	**584 (33.3)**	**1484 (46.5)**
**Employment condition**							
Formal	391 (54.19)	131 (12.1)	106 (11.7)	90 (12.4)	199 (27.5)	350 (53)	346 (26.2)
Informal	345 (45.81)	738 (87.9)	618 (88.3)	634 (87.6)	627 (72.5)	232 (47)	1131 (73.8)
**Labor relationship**							
Employee	373 (52.36)	280 (27.1)	284 (39.4)	289 (43.6)	271 (36.2)	355 (59.5)	855 (58.2)
Self-employed or employer	360 (47.64)	590 (72.9)	438 (60.6)	439 (56.4)	555 (63.8)	228 (40.5)	629 (41.8)
**Occupational category**							
Non-manual	491 (48.49)	560 (44.9)	527 (58.4)	398 (42.7)	554 (50.7)	438 (61.7)	996 (69)
Manual	246 (51.51)	310 (55.1)	198 (41.6)	330 (57.3)	272 (49.3)	146 (38.3)	488 (31)
**Age, years**							
under 24	89 (11.0)	79 (8.7)	185 (42.1)	114 (28.2)	96 (17.2)	76 (16)	363 (23.1)
25-44	358 (51.2)	409 (43.1)	351 (36.2)	374 (45.4)	427 (56.5)	312 (52.2)	694 (47.4)
45-64	260 (34.9)	305 (35.5)	148 (17.0)	211 (22.7)	256 (23.3)	170 (29.0)	326 (23.2)
≥65	30 (2.98)	77 (12.7)	41 (4.8)	29 (3.7)	47 (2.9)	26 (2.9)	101 (6.3)
**Education achieved**							
Less than primary school	11 (1.36)	56 (8.8)	72 (11.4)	36 (5.2)	70 (7.9)	5 (0.7)	120 (6.6)
Primary school	234 (33.8)	296 (38.3)	277 (38.9)	346 (48.8)	274 (32.8)	69 (15.7)	268 (17.2)
Secondary school	341 (47.2)	410 (42.9)	279 (39.1)	265 (36.8)	298 (40.2)	314 (51.7)	686 (48.2)
Higher education	151 (17.7)	108 (9.9)	97 (10.7)	81 (9.2)	184 (19.1)	196 (31.9)	410 (28)
**Marital status**							
Married or co-habiting	391 (53.2)	509 (59.3)	410 (54.5)	389 (51.4)	460 (54.3)	338 (56.4)	797 (52.6)
Single, divorced or widowed	345 (46.8)	361 (40.7)	315 (45.5)	339 (48.6)	365 (45.7)	243 (43.6)	686 (47.4)
**Total**	**1503**	**1374**	**1510**	**1507**	**1500**	**1505**	**3113**

WHO-5			Others		
**Argentina**	**Portugal**	**Spain**	**Chile**	**Brazil**	**Mexico**
**N (%)**	**N (%)**	**N (%)**	**N (%)**	**N (%)**	**N (%)**

**5035 (54.3)**	**396 (47.7)**	**1674 (53.2)**	**1529 (62.4)**	**29419 (53.7)**	**59836 (59.8)**
3050 (59.0)	250 (91.3)	1240 (96.1)	986 (73.1)	6314 (24.4)	19217 (50.6)
1985 (41.0)	27 (8.7)	54 (3.9)	391 (27.0)	23105 (75.6)	19916 (49.4)
3451 (68.4)	280 (73.1)	1308 (78.1)	1062 (77.1)	16539 (56.0)	43084 (72.0)
1584 (31.6)	116 (26.9)	364 (22.0)	314 (22.9)	12880 (44.0)	16274 (17.4)
2473 (48.0)	183 (48.6)	865 (56.5)	556 (52.2)	11969 (37.6)	-
2436 (52.0)	213 (51.4)	809 (43.5)	671 (47.8)	17450 (62.4)	-
504 (12.0)	16 (4.3)	99 (4.5)	89 (9.0)	2792 (4.2)	12426 (20.1)
2574 (50.1)	141 (41.8)	856 (53.1)	653 (47.8)	14143 (48.7)	26629 (44.9)
1715 (34.0)	206 (48.1)	707 (41.9)	679 (38.5)	10707 (42.2)	16988 (28.8)
242 (3.9)	31 (5.8)	9 (0.4)	108 (4.7)	1777 (4.9)	3793 (6.1)
289 (5.5)	8 (2.1)	53 (3.0)	177 (10.9)	10824 (38.3)	8444 (13.7)
1745 (37.2)	123 (26.1)	135 (7.3)	271 (17.7)	4606 (15.2)	11352 (18.8)
1651 (31.5)	184 (49.0)	629 (34.1)	665 (38.1)	9577 (31.4)	35387 (59.4)
1344 (25.8)	78 (22.8)	852 (55.7)	366 (33.3)	4412 (15.1)	4653 (8.2)
3215 (65.1)	286 (84.8)	1076 (76.6)	945 (65.0)	20599 (72.9)	39631 (66.3)
1814 (34.9)	53 (15.2)	353 (23.5)	568 (35.0)	8820 (27.1)	20205 (33.8)
**3931 (45.7)**	**500 (52.3)**	**1617 (46.8)**	**1597 (37.6)**	**22863 (46.3)**	**39851 (40.2)**
2230 (55.2)	352 (92.1)	1263 (93.2)	956 (70.4)	6150 (30.3)	12958 (55.4)
1701 (44.9)	41 (7.9)	106 (6.8)	409 (29.6)	16713 (69.7)	11056 (44.6)
2967 (74.9)	401 (84.6)	1396 (85.9)	1106 (83.2)	16035 (70.6)	27413 (69.2)
964 (25.1)	99 (15.4)	220 (14.1)	244 (16.8)	6828 (29.4)	11937 (22.2)
2845 (72.1)	328 (67.4)	1157 (76.4)	837 (71.6)	15086 (62.4)	-
1050 (27.9)	172 (32.6)	460 (23.6)	384 (28.4)	7777 (37.6)	-
294 (9.6)	15 (4.3)	87 (4.6)	104 (10.7)	2081 (3.7)	7137 (17.1)
2074 (50.2)	208 (46.3)	834 (54.1)	801 (52.0)	11504 (49.8)	19286 (48.6)
1415 (36.6)	245 (46.2)	676 (40.3)	631 (33.8)	8321 (42.7)	11614 (29.7)
148 (3.6)	32 (3.2)	18 (1.1)	61 (3.6)	957 (3.8)	1814 (4.6)
151 (3.7)	15 (1.7)	35 (2.0)	161 (10.4)	5377 (25.7)	4899 (12.5)
981 (26.2)	135 (23.7)	126 (7.2)	268 (18.1)	2927 (12.4)	6467 (16.6)
1157 (29.3)	226 (47.4)	557 (33.3)	687 (37.1)	8679 (35.9)	24971 (62.0)
1636 (40.9)	123 (27.3)	895 (57.6)	413 (34.4)	5880 (26)	3514 (9.0)
1840 (56.8)	334 (79.4)	977 (71.9)	767 (51.6)	12425 (57.6)	21503 (52.7)
2085 (43.2)	96 (20.6)	457 (28.1)	786 (48.4)	10438 (42.4)	18348 (47.3)
**8966**	**896**	**3291**	**3126**	**52282**	**99687**

GHQ-12: 12-item General Health Questionnaire. WHO-5: World Health Organization - Five Well-Being Index. Percentages are weighted by specific survey weights. Employment condition in Spain, Portugal and Mexico was only available for employees. Information on occupational category was not available for Mexico.

Supplementary Table S3. Prevalence of poor mental health [% (95% CI)] according to work and sociodemographic variables, by questionnaire, country, and sex.

	**Questionnaire and country**					*continues*
	**GHQ-12**					
**Men**	**Costa Rica**	**El Salvador**	**Guatemala**	**Honduras**	**Nicaragua**	**Panama**
**Total**	15.1 (10.7 - 19.6)	8.8 (5.4 - 12.3)	8.1 (5.9 - 11)	19.4 (16.3 - 22.9)	21 (17.3 - 25.1)	22.8 (19.0 - 27.0)
**Employment condition**[1]						
Formal	11.6 (6.5 - 16.7)	5.9 (2.1 - 9.7)	5.9 (2.0 - 16)	11.7 (6.5 - 20.1)	14.7 (9.2 - 22.6)	27.0 (22.0 - 32.7)
Informal	20.9 (12.7 - 29.1)	9.4 (5.3 - 13.5)	8.2 (5.9 - 11.3)	20.6 (17.1 - 24.6)	22.9 (18.6 - 28.0)	19.2 (14.1 - 25.7)
**Labor relationship**						
Employee	11.1 (5.9 - 16.3)	9.5 (4.5 - 14.5)	7.2 (4.0 - 12.9)	18.2 (13.3 - 24.3)	18.0 (12.8 - 24.7)	25.6 (20.0 - 32.1)
Self-employed or employer	19.6 (12.3 - 27)	8.5 (3.9 - 13.1)	8.5 (5.9 - 12.1)	20.4 (16.6 - 24.8)	23.2 (18.4 - 28.8)	20.7 (15.9 - 26.5)
**Occupational category**[2]						
Non-manual	14.6 (9.2 - 19.9)	10.1 (6.6 - 13.7)	3.7 (2.1 - 6.7)	12.7 (8.9 - 17.9)	10.9 (6.9 - 16.7)	21.5 (15.6 - 28.8)
Manual	15.3 (9.8 - 20.7)	8.6 (4.4 - 12.7)	9.8 (6.9 - 13.6)	20.8 (17.2 - 25.0)	23.5 (19.1 - 28.5)	23.1 (18.7 - 28.2)
**Age. years**						
Under 24	15.6 (0.7 - 30.4)	8.9 (3.1 - 14.6)	4.5 (1.9 - 10.1)	13.8 (7.0 - 25.4)	12.6 (6.3 - 23.5)	20.6 (12.5 - 32.0)
25-44	14.2 (7.8 - 20.6)	9.5 (4.6 - 14.4)	13.2 (9.0 - 19.0)	15 (11.2 - 19.6)	21.5 (16.5 - 27.4)	22.7 (17.7 - 28.8)
45-64	19.4 (11.1 - 27.6)	9.3 (2.6 - 15.9)	5.2 (2.4 - 10.6)	26.7 (21.1 - 33.1)	27.6 (20.4 - 36.2)	24.0 (17.1 - 32.5)
≥ 65	2.2 (0.1 - 4.3)	7.0 (-2.9 - 16.9)	7.8 (2.5 - 21.5)	30.8 (21.5 - 42.0)	19.2 (8.1 - 39.3)	22.8 (11.2 - 41.0)
**Education**						
Less than primary school	8.1 (-2.1 - 18.2)	13.3 (-1.6 - 28.1)	13.3 (5.9 - 27.4)	32.9 (18.4 - 51.7)	37.1 (25.1 - 51.0)	19.4 (5.6 - 49.3)
Primary school	18.9 (11.6 - 26.3)	10.1 (3.0 - 17.3)	8.6 (5.6 - 13.1)	20.8 (17.0 - 25.3)	18.3 (12.9 - 25.4)	21.1 (13.7 - 31.2)
Secondary school	12.9 (6 - 19.7)	6.6 (3.9 - 9.3)	7.1 (4.0 - 12.2)	15.0 (10.4 - 21.1)	21.0 (15.1 - 28.4)	23.9 (19.2 - 29.4)
Higher education	7.7 (1.9 - 13.6)	8.5 (2.2 - 14.7)	2.9 (0.9 - 9.1)	4.5 (1.7 - 11.7)	15.2 (8.6 - 25.5)	22.4 (14.3 - 33.2)
**Marital status**						
Married or co-habiting	15.3 (9.6 - 21.1)	5.9 (2.4 - 9.4)	8.0 (5.3 - 11.7)	21.1 (17.7 - 25.1)	21.7 (17.1 - 27.2)	22.3 (17.5 - 27.9)
Single, divorced or widowed	14.7 (7.8 - 21.6)	14 (6.8 - 21.1)	8.3 (5.0 - 13.4)	15.7 (10.1 - 23.7)	19.2 (13.8 - 26.1)	23.4 (17.8 - 30.1)
**Women**	**Costa Rica**	**El Salvador**	**Guatemala**	**Honduras**	**Nicaragua**	**Panama**
**Total**	14.8 (11.0 - 18.7)	11 (8.5 - 13.4)	13.4 (10.2 - 17.4)	23.2 (19.4 - 27.5)	21.4 (17.7 - 25.5)	25.3 (20.7 - 30.7)
**Employment condition**[1]						
Formal	14.3 (9.2 - 19.4)	14.5 (8.7 - 20.3)	7.3 (3.2 - 15.5)	18.4 (10.6 - 29.9)	19.0 (12.5 - 27.9)	24.1 (19.3 - 29.8)
Informal	15.4 (9.6 - 21.2)	10.5 (7.9 - 13.2)	14.2 (10.7 - 18.7)	23.9 (19.8 - 28.7)	22.3 (18.1 - 27.1)	26.8 (18.9 - 36.6)
**Labor relationship**						
Employee	12.5 (7.8 - 17.3)	13.9 (9.4 - 18.4)	9.7 (5.3 - 17.0)	21.6 (15.3 - 29.6)	21.7 (15.5 - 29.6)	24.0 (18 - 31.2)
Self-employed or employer	17 (10.9 - 23.1)	9.9 (7.0 - 12.8)	15.2 (11.2 - 20.4)	24.4 (20.1 - 29.3)	21.1 (16.9 - 26.2)	27.8 (20.7 - 36.2)
**Occupational category**[2]						
Non-manual	16.2 (11.2 - 21.2)	13 (10 - 16.1)	10.5 (7.5 - 14.5)	25.2 (19.3 - 32.2)	17.4 (13.9 - 21.4)	23.2 (19.1 - 27.9)
Manual	13.5 (7.7 - 19.2)	9.3 (5.7 - 13)	17.4 (11.4 - 25.7)	21.7 (17.0 - 27.2)	25.5 (19.2 - 32.9)	28.8 (19.1 - 40.8)
**Age. years**						
under 24	7.3 (2.5 - 12.1)	11.6 (3.3 - 19.9)	9.5 (5.0 - 17.4)	19.9 (11.2 - 32.7)	21.5 (11.9 - 35.9)	19.7 (10.8 - 33.2)
25-44	17.1 (10.9 - 23.2)	12.5 (8.9 - 16.2)	15.9 (11.3 - 21.9)	22.6 (18.4 - 27.4)	20.8 (16.1 - 26.4)	26.1 (19.5 - 33.9)
45-64	14.1 (8.1 - 20.2)	8.7 (5.0 - 12.4)	16.7 (9.8 - 27.1)	24.8 (18.6 - 32.3)	22.4 (16.4 - 29.7)	24.3 (17.3 - 33.2)
≥65	11.5 (0.3 - 22.6)	11.7 (3.1 - 20.3)	16.5 (5.1 - 41.7)	45.2 (26.4 - 65.5)	23.3 (10.4 - 44.3)	53.7 (25.6 - 79.7)
**Education**						
Less than primary school	56.0 (17.9 - 94.1)	16.5 (3.7 - 29.3)	26.8 (15.2 - 42.9)	48.2 (30.5 - 66.4)	37.7 (24.6 - 53.0)	36.5 (8.7 - 77.5)
Primary school	11.3 (6.1 - 16.5)	8.5 (5.0 - 11.9)	13.5 (8.7 - 20.4)	27.6 (21.4 - 34.7)	24.1 (17.5 - 32.2)	35.7 (19.2 - 56.5)
Secondary school	17.2 (11.0 - 23.4)	11.4 (7.7 - 15.1)	11.6 (7.1 - 18.4)	17.2 (12.4 - 23.4)	19.5 (13.9 - 26.5)	25.3 (19.7 - 31.9)
Higher education	11.8 (3.7 - 20.0)	14.2 (7.8 - 20.5)	5.0 (2.1 - 11.5)	9.3 (4.9 - 17.0)	13.9 (8.7 - 21.4)	20.0 (13.9 - 28.0)

**Marital status**						
Married or co-habiting	13.8 (8.5 - 19.1)	10.3 (6.9 - 13.6)	14.8 (10.5 - 20.4)	23.4 (18.9 - 28.6)	20.9 (16.1 - 26.8)	25.1 (19.1 - 32.3)
Single, divorced or widowed	16 (10.3 - 21.6)	12.0 (8.5 - 15.6)	11.7 (7.3 - 18.0)	23.0 (17.1 - 30.2)	21.9 (16.8 - 28.2)	25.7 (18.8 - 34.0)

**Questionnaire and country**						
**GHQ-12**	**WHO-5**			**Other**		
**Peru**	**Argentina**	**Portugal**	**Spain**	**Chile**	**Brazil**	**Mexico**
27.9 (25.5 - 30.4)	15.6 (14.4 - 16.8)	11.6 (8.4 - 15.8)	9.9 (8.4 - 11.8)	15.8 (13.3 - 18.6)	4.6 (4.2 - 5.0)	3.7 (3.5 - 3.9)
20.5 (16.7 - 24.3)	15.5 (13.5 - 17.5)	11.1 (7.4 - 16.4)	9.8 (8.0 - 12)	15.1 (12.1 - 18.8)	4.8 (4.0 - 5.8)	3.0 (2.6 - 3.4)
32.3 (29.1 - 35.4)	15.7 (14.0 - 17.5)	10.7 (3.1 - 30.7)	19.7 (9.1 - 37.4)	17.1 (12.4 - 23.0)	4.5 (4.1 - 5.0)	3.5 (3.1 - 3.9)
25.9 (22.8 - 29.1)	14.7 (13.0 - 16.3)	11.2 (7.7 - 16.1)	10.2 (8.3 - 12.3)	16.6 (13.6 - 20.2)	4.3 (3.8 - 4.8)	3.2 (2.9 - 3.4)
30.8 (27.0 - 34.6)	17.6 (15.6 - 19.7)	12.7 (6.8 - 22.6)	9.3 (6.3 - 13.4)	13.0 (8.6 - 19.1)	4.9 (4.3 - 5.6)	5.2 (4.6 - 5.8)
23.3 (20.1 - 26.8)	15.6 (14.3 - 17.0)	9.1 (5.2 - 15.4)	9.9 (7.7 - 12.6)	12.3 (8.8 - 16.9)	5.5 (4.8 - 6.2)	-
32.3 (28.9 - 35.9)	15.8 (13.3 - 18.2)	14.1 (9.5 - 20.4)	10.0 (7.8 - 12.8)	17.8 (14.0 - 22.5)	4.0 (3.6 - 4.5)	-
23.6 (19.1 - 28.9)	13.6 (7.2 - 20.0)	4.6 (0.6 - 27.7)	4.9 (2.1 - 11.0)	7.5 (3.5 - 15.3)	4.4 (3.3 - 5.8)	1.4 (1.1 - 1.8)
23.6 (20.2 - 27.4)	14.9 (13.5 - 16.4)	11.8 (6.9 - 19.2)	10.2 (7.9 - 12.9)	14.8 (11.2 - 19.5)	4.4 (3.9 - 4.9)	3.4 (3.1 - 3.7)
32.6 (27.8 - 37.8)	18.1 (16.2 - 20.0)	12.8 (8.3 - 19.3)	10.4 (8.0 - 13.3)	17.8 (14.1 - 22.2)	5.0 (4.3 - 5.8)	5.3 (4.8 - 5.8)
46.5 (37.8 - 55.5)	8.2 (3.9 - 12.5)	-	-	24.9 (15.2 - 38.0)	3.3 (2.2 - 4.8)	6.2 (5.2 - 7.4)
45.7 (34.6 - 57.3)	18.6 (11.2 - 25.9)	15.6 (2.2 - 60.3)	20.3 (9.5 - 38.2)	28.5 (20.1 - 38.7)	4.5 (3.9 - 5.2)	5.0 (4.4 - 5.6)
41.3 (34.7 - 48.3)	16.2 (14.2 - 18.1)	14.7 (8.2 - 24.8)	8.9 (4.7 - 16.1)	16.7 (11.6 - 23.3)	4.6 (3.6 - 5.7)	3.9 (3.4 - 4.4)
26.5 (23.2 - 30.0)	13.5 (11.8 - 15.2)	13.3 (8.6 - 19.9)	9.0 (6.7 - 12.1)	15.7 (12.0 - 20.3)	4.6 (3.9 - 5.3)	3.3 (3.0 - 3.6)
21.5 (17.6 - 26.0)	16.7 (14.7 - 18.7)	3.4 (1.2 - 9.4)	10.1 (8 - 12.8)	10.8 (6.8 - 16.7)	4.7 (3.8 - 5.8)	4.1 (3.3 - 5.1)
29.0 (25.8 - 32.4)	15.6 (14.3 - 16.9)	12.2 (8.4 - 17.3)	10.6 (8.5 - 13.0)	14.6 (11.8 - 17.9)	4.1 (3.7 - 4.6)	4.2 (4.0 - 4.5)
26.6 (23.1 - 30.4)	15.6 (13.5 - 17.7)	11.4 (4.7 - 25.3)	5.8 (3.7 - 9.0)	17.4 (12.9 - 22.9)	5.9 (5.1 - 6.7)	2.6 (2.3 - 3.0)
**Peru**	**Argentina**	**Portugal**	**Spain**	**Chile**	**Brazil**	**Mexico**
32.5 (29.9 - 35.3)	21.9 (20.2 - 23.5)	14.1 (11.1 - 17.7)	13.7 (11.8 - 15.9)	21.8 (19.0 - 25.0)	13.3 (12.6 - 14.1)	6.5 (6.2 - 6.9)
24.3 (19.1 - 29.5)	21.4 (19.7 - 23.1)	12.9 (9.6 - 17.1)	12.4 (10.4 - 14.8)	18.9 (15.6 - 22.8)	11.9 (10.6 - 13.4)	4.6 (4.1 - 5.1)
35.3 (32.2 - 38.5)	22.4 (19.0 - 25.8)	21.9 (10.3 - 40.7)	18.3 (11.0 - 29.0)	28.5 (21.9 - 36.2)	13.9 (13.1 - 14.8)	7.1 (6.4 - 7.8)
27.3 (24.0 - 30.6)	20.6 (17.6 - 23.6)	13.5 (10.3 - 17.6)	12.9 (10.9 - 15.2)	20.3 (17.1 - 24.1)	12.9 (12.0 - 13.8)	5.5 (5.1 - 5.9)
39.8 (35.5 - 44.2)	25.7 (13.2 - 38.1)	17.1 (10.7 - 26.2)	18.9 (13.3 - 26.1)	28.5 (19.9 - 39.0)	14.4 (13.0 - 15.9)	9.1 (8.2 - 10.0)
28.9 (25.8 - 32.1)	20.7 (19.0 - 22.3)	11.5 (8.3 - 15.7)	13.5 (11.4 - 16.0)	19.8 (16.1 - 24.3)	13.1 (12.2 - 14.0)	-
40.8 (36.0 - 45.9)	25.3 (18.1 - 32.5)	19.5 (13.7 - 26.9)	14.5 (10.8 - 19.2)	27.9 (21.5 - 35.4)	13.7 (12.5 - 15.0)	-
25.5 (20.8 - 30.7)	24.3 (13.1 - 35.5)	15.7 (3.8 - 47.0)	2.7 (0.5 - 12.7)	16.4 (8.5 - 29.2)	15.5 (13.3 - 18.0)	2.3 (1.9 - 2.8)
30.6 (26.9 - 34.6)	22.7 (21.1 - 24.4)	7.9 (5.0 - 12.3)	12.4 (9.9 - 15.4)	17.2 (13.6 - 21.4)	13.1 (12.1 - 14.1)	5.5 (5.1 - 6.0)
37.1 (31.3 - 43.2)	21.0 (18.7 - 23.2)	19.0 (14.1 - 25.1)	16.8 (13.6 - 20.6)	30.9 (25.8 - 36.4)	13.8 (12.6 - 15.1)	9.9 (9.1 - 10.7)
55.6 (44.5 - 66.1)	13.1 (7.3 - 18.9)	30.5 (15.4 - 51.4)	15.0 (4.6 - 39.4)	21.1 (11.5 - 35.7)	8.9 (6.4 - 12.2)	11.8 (10.0 - 13.8)
51.1 (41.1 - 60.9)	26.2 (14.6 - 37.7)	47.7 (23.3 - 73.3)	20.2 (9.0 - 39.1)	38.9 (28.0 - 51.0)	15.5 (14.0 - 17.1)	10.8 (9.7 - 12.1)

41.3 (34.9 - 48.1)	21.8 (19.0 - 24.7)	20.1 (13.4 - 28.9)	27.4 (18.6 - 38.4)	27.3 (20.7 - 35.0)	15.5 (13.4 - 17.8)	8.3 (7.4 - 9.3)
32.6 (28.7 - 36.7)	24.8 (16.4 - 33.2)	14.1 (9.8 - 19.7)	11.7 (9.1 - 15.1)	19.9 (15.8 - 24.6)	12.3 (11.2 - 13.6)	5.4 (5.0 - 5.8)
22.8 (18.7 - 27.5)	19.5 (14.1 - 24.9)	7.1 (3.7 - 13.3)	13.0 (10.5 - 16.0)	16.9 (12.1 - 23.0)	11.5 (10.2 - 13.0)	5.2 (4.1 - 6.5)
30.7 (27.3 - 34.3)	21.3 (19.4 - 23.1)	13.1 (9.7 - 17.5)	13.2 (10.8 - 16)	20.4 (16.7 - 24.6)	12.6 (11.6 - 13.6)	6.2 (5.8 - 6.7)
34.5 (30.5 - 38.6)	22.8 (20.0 - 25.5)	19.4 (12.1 - 29.5)	14.5 (11.0 - 18.8)	22.3 (18.0 - 27.2)	14.4 (13.2 - 15.5)	6.9 (6.4 - 7.5)

GHQ-12: 12-item General Health Questionnaire. WHO-5: World Health Organization - Five Well-Being Index. Percentages are weighted by specific survey weights. Employment condition in Spain, Portugal and Mexico was only available for employees. Information on occupational category was not available for Mexico.
